# Characterization and comparison of novel adjuvants for a prefusion clamped MERS vaccine

**DOI:** 10.3389/fimmu.2022.976968

**Published:** 2022-09-02

**Authors:** Jake S. O’Donnell, Ariel Isaacs, Virginie Jakob, Celia Lebas, James B. Barnes, Patrick C. Reading, Paul R. Young, Daniel Watterson, Patrice M. Dubois, Nicolas Collin, Keith J. Chappell

**Affiliations:** ^1^ The Australian Institute for Bioengineering and Nanotechnology, The University of Queensland, Brisbane, QLD, Australia; ^2^ The School of Chemistry and Molecular Biosciences, The University of Queensland, Brisbane, QLD, Australia; ^3^ Vaccine Formulation Institute, Geneva, Switzerland; ^4^ The WHO Collaborating Centre for Reference and Research on Influenza, Peter Doherty Institute for Infection and Immunity, Melbourne, VIC, Australia; ^5^ Department of Microbiology and Immunology, The University of Melbourne, Peter Doherty Institute for Infection and Immunity, Melbourne, VIC, Australia; ^6^ Australian Infectious Disease Research Centre, The University of Queensland, Brisbane, QLD, Australia

**Keywords:** MERS, vaccine, adjuvant, antibody, T cell

## Abstract

Various chemical adjuvants are available to augment immune responses to non-replicative, subunit vaccines. Optimized adjuvant selection can ensure that vaccine-induced immune responses protect against the diversity of pathogen-associated infection routes, mechanisms of infectious spread, and pathways of immune evasion. In this study, we compare the immune response of mice to a subunit vaccine of Middle Eastern respiratory syndrome coronavirus (MERS-CoV) spike protein, stabilized in its prefusion conformation by a proprietary molecular clamp (MERS SClamp) alone or formulated with one of six adjuvants: either (*i*) aluminium hydroxide, (*ii*) SWE, a squalene-in-water emulsion, (*iii*) SQ, a squalene-in-water emulsion containing QS21 saponin, (*iv*) SMQ, a squalene-in-water emulsion containing QS21 and a synthetic toll-like receptor 4 (TLR4) agonist 3D-6-acyl Phosphorylated HexaAcyl Disaccharide (3D6AP); (*v*) LQ, neutral liposomes containing cholesterol, 1.2-dioleoyl-sn-glycero-3-phosphocholine (DOPC) and QS21, (*vi*) or LMQ, neutral liposomes containing cholesterol, DOPC, QS21, and 3D6AP. All adjuvanted formulations induced elevated antibody titers which where greatest for QS21-containing formulations. These had elevated neutralization capacity and induced higher frequencies of IFN_Ɣ_ and IL-2-producing CD4^+^ and CD8^+^ T cells. Additionally, LMQ-containing formulations skewed the antibody response towards IgG2b/c isotypes, allowing for antibody-dependent cellular cytotoxicity. This study highlights the utility of side-by-side adjuvant comparisons in vaccine development.

## Summary

This study presents a side-by-side comparison of immunological responses in mice to a subunit vaccine when separately formulated with a panel of 6 different adjuvants. Individual pairings induced immune responses with unique features including differences in antibody titer, antibody isotype, and T cell characteristics.

## Introduction

Vaccines play a critical role in controlling the spread of diseases and in reducing their severity (1). By exposing the immune system to pathogen-associated antigens, vaccines can enable the development of immunological memory and protective immunity, without the danger of natural infection (1). Various vaccine modalities have been developed including inactivated pathogens, live-attenuated pathogens, toxoids, conjugates, DNA, mRNA, and protein subunits (1). Of these, protein subunit vaccines offer many advantages including superior safety profiles and the ability to rationally design specific structural conformations capable of mimicking native pathogen structures; a critical factor for raising broadly-neutralising antibody responses (2).

To trigger immune activation, non-replicative subunit vaccines are formulated to include chemical compounds capable of stimulating immune activation, known as adjuvants (3). Many adjuvants have been developed, capable of increasing the magnitude, breadth, and durability of the antigen-specific immune response (3). Aluminium salts are the most common adjuvant used in vaccines, with a strong safety profile shown following billions of doses in humans (4). It is thought to act through a combination of local inflammation, the depot effect, and increased uptake of antigen by antigen presenting cells (APCs), leading to a T-helper 2 (TH2)-biased immune response (4). Squalene oil-in-water emulsions, such as MF59^®^ (Seqirus) or AS03 (GSK), are also used in several vaccines. These compounds recruit immune cells to the injection site by inducing chemokines, enhancing cross-presentation of antigens by APCs to activate antigen-specific T cells (5). QS21 is a saponin which can trigger NLRP3 inflammasome in mouse APCs and subsequent release of caspase-1-dependent cytokines, IL-1β and IL-18 (6). 3D-6-acyl Phosphorylated HexaAcyl Disaccharide (3D6AP) is a synthetic TLR4 agonist that resembles the bacterial Monophophoryl Lipid A toll-like receptor 4 (TLR4) agonist 3D-MPL capable of raising Th1-biased immune responses (7). Liposomes containing 1.2-dioleoyl-sn-glycero-3-phosphocholine (DOPC) have also demonstrated efficacy as adjuvant components by preferentially interacting with APCs to enhance their exposure to adjuvant and stimulate activation (8).

For most researchers and vaccine developers, access to new generation adjuvants remains a major vaccine R&D challenge as most clinically tested new generation adjuvants are owned by a few vaccine manufacturers. This hampers vaccine research and development and limits equitable access to vaccines especially in low- and middle-income countries. The adjuvants included in this study are available under an open-access model and are, or will become soon, available at clinical grade to enable clinical evaluation of novel candidate vaccines.

We have previously developed a subunit vaccine of Middle Eastern respiratory syndrome coronavirus (MERS-CoV, referred to throughout as MERS) spike protein, stabilized in its prefusion conformation by a proprietary molecular clamp (MERS SClamp), which was shown to elicit potent neutralising antibodies (9–11). Given that induction of potent humoral and cellular immunity is likely required for a sustained protective immune response against MERS-CoV, we conducted a comprehensive analysis of MERS SClamp-induced immunity and investigated the effects of different adjuvant pairings. Here, we selected a panel of adjuvants including: (i) aluminium hydroxide (AlOH/Alhydrogel; Croda) (3, 12), (ii) SWE, a squalene-in-water emulsion (SWE; Seppic) (13, 14), (iii) SQ, a squalene-in-water emulsion containing the QS21 saponin (Vaccine Formulation Institute [VFI]), (iv) SMQ, a squalene-in-water emulsion containing QS 21 and 3D6AP (15), (v) LQ, neutral liposomes containing cholesterol, DOPC, and QS21 (VFI), (vi) or LMQ, neutral liposomes containing cholesterol, DOPC, QS21 and 3D6AP (VFI) (16). We assessed both humoral and cellular immune responses in mice, including neutralising antibodies, antibody isotypes, antibody-dependent cellular cytotoxicity (ADCC), and the activation and function of CD4^+^ and CD8^+^ T cells. To our knowledge, this is the most comprehensive immune analysis of any subunit vaccine to date.

## Results

### MERS SClamp-specific IgG titers vary depending on antigen-adjuvant combination

**Figure 1:**
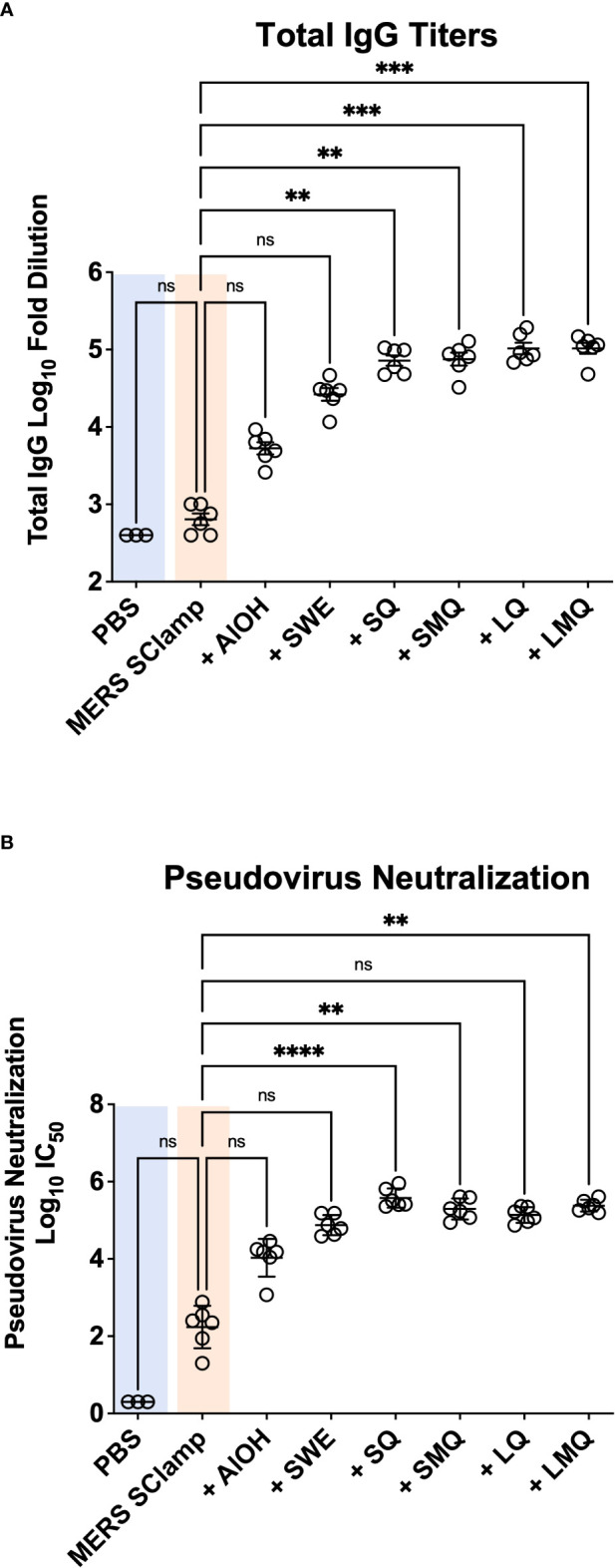
Vaccine adjuvanticity impacts antibody titer and neutralization. Following immunization of C57BL/6 mice with either PBS or clamped MERS antigen (MERS SClamp) (1µg/dose) +/- either AlOH salts, SWE, SQ, SMQ, LQ, or LMQ adjuvants **(A)** ELISAs showing anti-MERS IgG titer of serum from treated mice. **(B)** From the same experiment as **(A)**, IC_50_ of MERS pseudovirus neutralization with anti-MERS IgG of serum from mice treated with either MERS SClamp antigen +/- adjuvants. Individual data point have been presented with mean +/- SEM. Kruskal-Wallis with Dunn’s test, ns = P>0.05, **P<0.01, ***P<0.001, and ****P<0.0001. Experiment completed once, n = 3 – 6 mice/group. Related to **Figure S1**.

Vaccine adjuvants can differentially impact the magnitude and efficacy of immune responses directed towards immunizing antigens. We first performed an immunization study in which our previously developed MERS antigen (MERS SClamp) (9) (1 µg/dose) was formulated with or without either AlOH, SWE, SQ, SMQ, LQ, or LMQ adjuvants. Formulations were checked for adjuvant and antigen integrity (**Table S1**), then administered to C57BL/6 mice on day 0 and 21. All vaccinations were well tolerated, and no adverse events were observed. To evaluate correlates of protective immunity, we chose not to focus on the induction of IgA and mucosal immunity, as these are generally poor following standard intramuscular vaccination. Instead, we focused on serum IgG which is well documented to provide strong protection against infection in humans (17). On day 35, IgG antibody titers were measured in serum of each mouse by ELISA (**Figure 1A**). The inclusion of either AlOH or SWE showed a strong trend towards higher MERS SClamp-specific antibody titers above that seen following immunization with MERS SClamp alone (AlOH = 8.3-fold, *P* = >0.99; SWE = 41.5-fold, *P* = 0.66). The inclusion of the QS21-based adjuvants, SQ, SMQ, LQ, and LMQ, further increased antibody titers, by roughly 3.9 – 2.7-fold compared to SWE (**Figure 1A**). To test the effect that antigen dose had on MERS SClamp-specific antibody titer, a separate study was performed in parallel over the same time course in which MERS SClamp was formulated at a higher dose (5 µg/dose) with the same adjuvant groups. The antibody titers for each condition between the low and high-dose studies were comparable (**Figure S1A**). As such the low-dose schedule was used in subsequent studies.

In the context of viral pathogens, a critical feature of an effective vaccine-induced immune response is the production of virus-specific antibodies capable of neutralizing virus to limit infectious spread. We next tested the capacity of immunized serum from mice in each condition to neutralize MERS pseudovirus *in vitro* (**Figure 1B**). Pseudovirus neutralization was seen for all groups immunized with MERS SClamp, including non-formulated MERS SClamp in the absence of any adjuvant. The highest level of pseudovirus neutralisation was again observed for the QS21-containing adjuvant formulations, SQ, SMQ, LQ, and LMQ (**Figure 1C**). A strong trend was seen in which greater overall IgG titers correlated with higher neutralization capacity (r^2^ = 0.881, *P* = <0.0001) (**Figure S1B**).

### Vaccine-induced antibody responses vary with potential for ADCC induction

**Figure 2:**
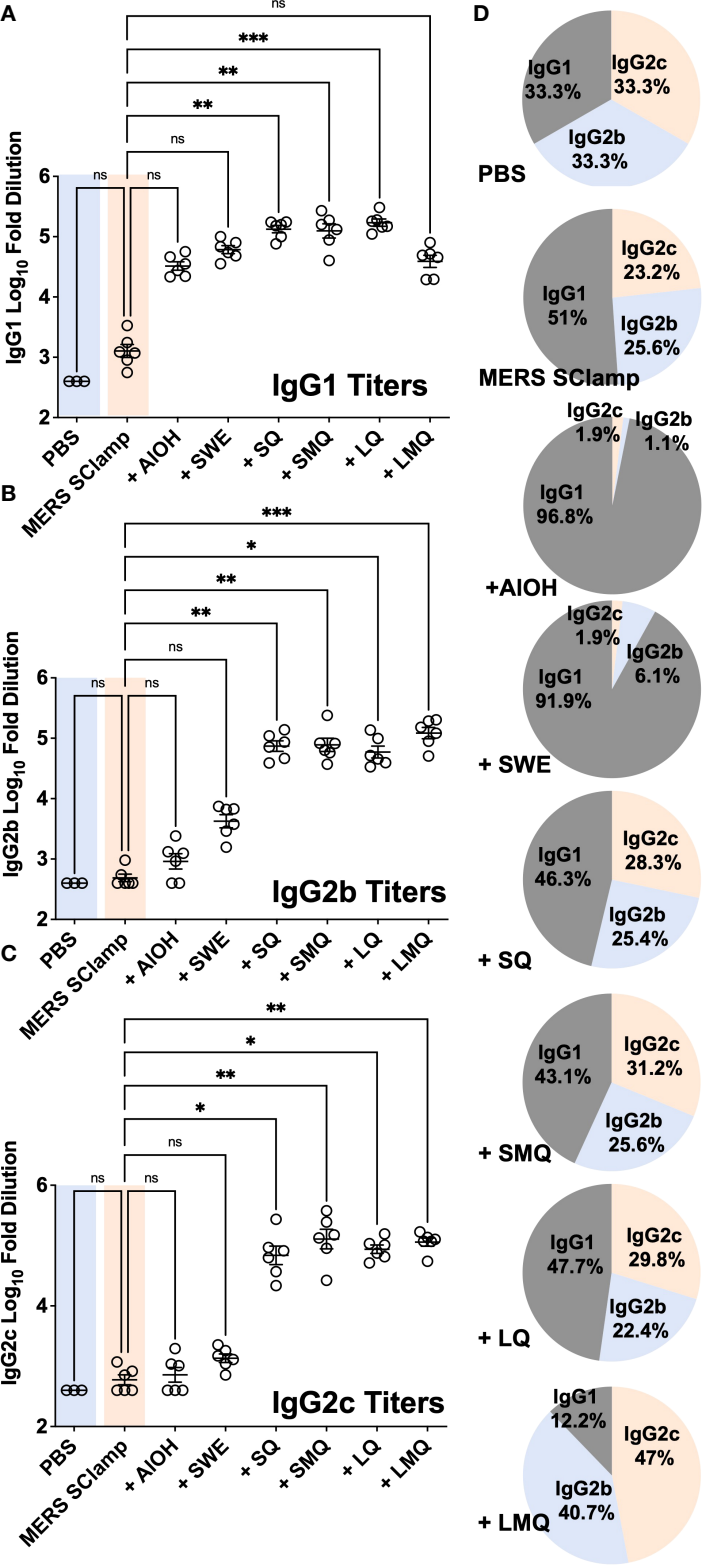
Tested adjuvants elicit different antibody isotypes. Mouse serum from the same experiment as **Figure 1A** was assessed for antibody isotype by ELISA with secondary antibodies specific for mouse IgG1 **(A)**, IgG2b **(B)**, IgG2c **(C)**, and **(D)** proportion summaries of IgG isotypes induced by each adjuvant. Individual data points presented with mean +/- SEM. Kruskal-Wallis with Dunn’s test, ns = P>0.05, *P<0.05, **P<0.01, and ***P<0.001. Experiment completed once, n = 3 – 6 mice/group.

In response to either natural infection or effective vaccination, antibody responses can be induced, capable of triggering immune-mediated clearance of virally infected cells expressing target antigens (18, 19). This process, termed antibody-dependent cellular cytotoxicity (ADCC) is primarily mediated by natural killer (NK) cells and other immune cells which express receptors capable of recognizing and binding target-bound antibodies *via* their fraction crystallizable (Fc) domains (20). This process depends on the isotype of antibodies produced during the immune response. For example, in humans IgG1 and in mice IgG2a or the analogous IgG2c antibodies are key players in facilitating ADCC (21–23). Therefore, we next aimed to understand whether immunization with MERS SClamp formulated with different adjuvants would affect the isotype of the antibody response (**Figures 2A**–**D**). In comparison to MERS SClamp-immunized mice, IgG1 titers were elevated by a similar amount following immunization with all formulations (**Figure 2A**). However, for IgG2b and IgG2c, QS21-containing formulations, +SQ, +SMQ, +LQ, and +LMQ elevated titres by greater than 10-fold and 100-fold, respectively, compared to formulations lacking QS21 (**Figures 2B**, **2C**). Analysis of isotype proportion revealed striking differences in isotype biasing between each adjuvant formulation with AlOH, SWE, SQ, SMQ, and LQ favouring IgG1, and LMQ adjuvant favouring IgG2b/c (**Figure 2D**).

Given that the tested adjuvants had differing capacity to induce IgG2c antibodies, we expected the QS21-containing formulations to most effectively trigger ADCC of cells infected with or expressing MERS antigen. To test this, we adapted a flow cytometry-based *in vitro* ADCC assay in which we measured the capacity of immunized serum from each condition to induce cell death of a cell line expressing MERS spike (target cells) when co-cultured with mouse splenocytes (effector cells) (24). For this, we engineered the LA-4 mouse cell line to stably express MERS antigen (LA-4 MERS) and combined it with MERS SClamp-immunized serum. Plasma membrane binding of antibodies in MERS-immunized serum was first confirmed by flow cytometry (**Figures S3A**, **B**).

**Figure 3:**
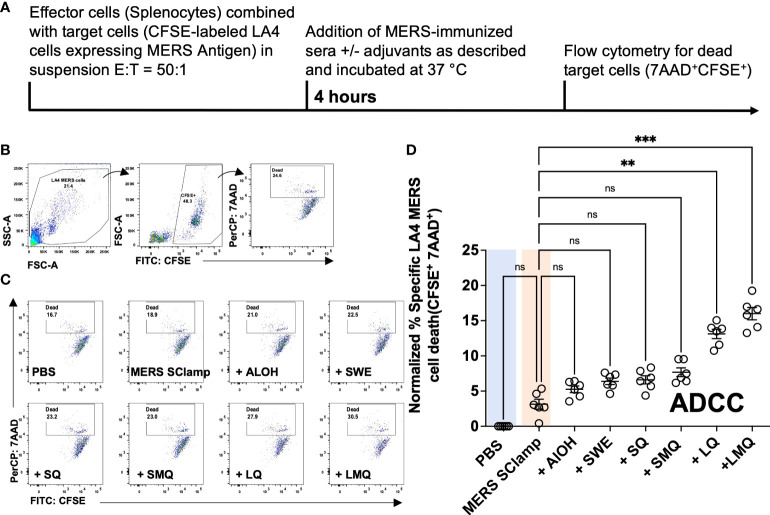
Vaccine-induced antibody responses vary with potential for ADCC induction. **(A)** ADCC assay experimental protocol. **(B)** Gating strategy for ADCC assay in which CFSE-labeled mouse LA4 MERS cells were incubated with BALB/c splenocytes (E:T = 50:1) and serum from mice immunized with MERS SClamp antigen +/- indicated adjuvants for 4-hours. Gating on live cells of LA-4 cell morphology, cell death (7AAD^+^) was measured among CFSE^+^ cells. **(C)** Representative flow cytometry plots of the proportion of dead LA4 MERS cells (CFSE^+^7AAD^+^) for each condition. **(D)** Data summary, where experimental data have been normalized to the mean percentage cell death of the PBS control condition. Data presented as normalized individual data points with mean +/- SEM. Kruskal-Wallis with Dunn’s test, ns = P>0.05, **P<0.01, ***P<0.001. Experiment completed once, n = 6 mice/group. Related to **Figure S2**.

Next, the ADCC assay was performed with an effector to target cell ratio (E:T) of 50:1, with mouse serum samples diluted to 1/10^3^ (**Figure 3A**). After 4 hours of incubation, cell death was measured by flow cytometry (**Figures 3A**–**D**). When normalized to background cell death seen in the condition incubated with PBS-immunized serum, a significant increase in the percentage of cell death was only seen for groups with formulations including liposomes; +LQ and +LMQ (**Figure 3D**). Importantly, ADCC capacity correlated with IgG2c titers (r^2^ = 0.516, *P* = <0.0001) (**Figure S3C**).

### Adjuvant selection can impact functional characteristics of the vaccine-specific T cell response

**Figure 4:**
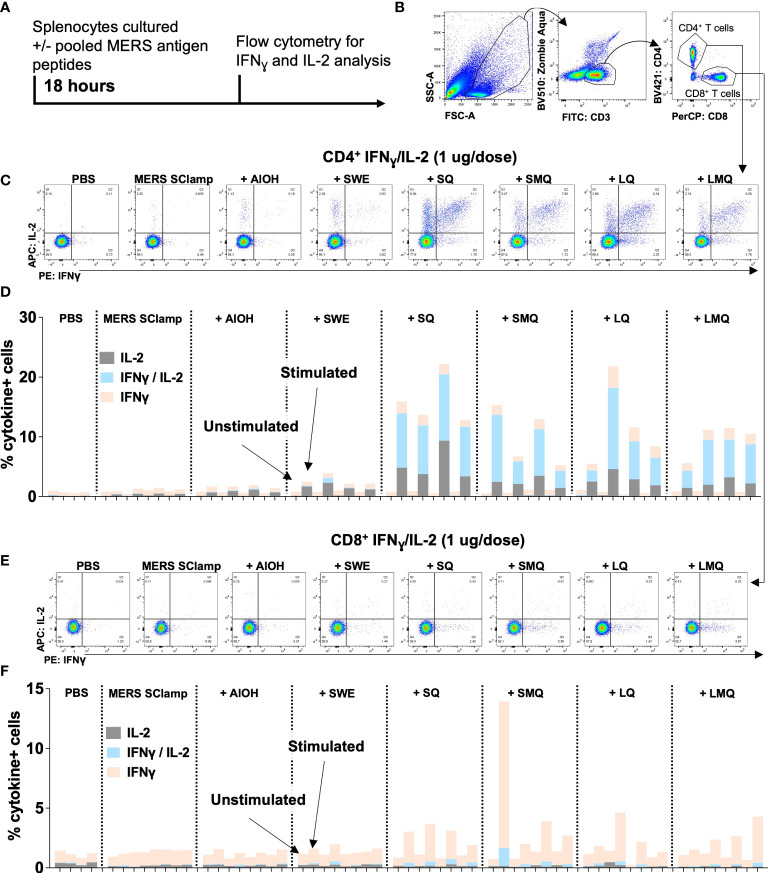
CD4^+^ and CD8^+^ T cell function differs following MERS SClamp immunization depending on adjuvant used. **(A)** Splenocytes were isolated from C57/BL6 mice immunized according to **Figure 1A**, incubated with or without pooled MERS SClamp antigen peptides for 4 hours in the presence of protein transport inhibitors followed by flow cytometry analysis. **(B)** Gating on live cells of lymphocyte morphology, T cells (CD3^+^CD8^+^ or CD3^+^CD4^+^) were assessed for expression of interferon gamma (IFN_Ɣ_) and interleukin-2 (IL-2). **(C)** Representative flow cytometry plots of IFN_Ɣ_ and IL-2 by CD4^+^ T cells treated as described in **(A)**, and **(D)** Data summary. **(E)** Representative flow cytometry plots of IFN_Ɣ_ and IL-2 by CD4^+^ T cells treated as described in **(A)**, and **(F)** Data summary. Individual data points for each mouse have been presented, n = 2 – 4/group. Related to **Figure S3**.

A critical component of the adaptive immune response following vaccination is the activation of vaccine antigen-specific T cells. Of these, CD4^+^ T cells provide help to activate B cells which produce vaccine antigen-specific antibodies, and cytotoxic CD8^+^ T cells capable of killing virus-infected cells. To test what impact each of the vaccination conditions had on the MERS SClamp-specific T cell response, splenocytes were collected from immunized mice and cultured ex vivo with or without a pool of MERS Spike ectodomain 20mer peptides for 18 hours (**Figure 4A**). Following incubation, flow cytometry was used to evaluate T cell markers and intracellular expression of IL-2 and IFN_Ɣ_ (**Figure 4B**). Among CD4^+^ T cells, cytokine expression by unstimulated cells was negligible, however, the proportion of IFN_Ɣ_^+^IL-2^+^ was greater for experimental groups including QS21 (**Figures 4C**, **D**). Interestingly, this was greatest for mice immunized with MERS SClamp +SQ adjuvant (**Figure 4C**, **D**) and was further elevated in samples from mice immunized with a higher antigen dose (**Figure S3A**). IFN_Ɣ_^+^ CD8^+^ T cells were detected at low frequency following stimulation for experimental groups immunized with formulations containing QS21, and IL-2^+^ were very rarely detected (**Figure 4E**, **F**). Interestingly, when immunized with a higher antigen dose, the proportion of IFN_Ɣ_^+^ CD8^+^ were more frequent for all experimental groups immunized with formulations containing QS21 (**Figure S3B**).

## Discussion

In this study we have compared features of immunological responses in mice to a subunit vaccine when separately formulated with a panel of 6 different adjuvants. In doing so, we have demonstrated that individual pairings can induce immune responses with unique features including differences in antibody titer, antibody isotype, and T cell frequency. Consequently, the ability of vaccine antigen-specific antibodies to induce viral neutralisation or ADCC, or of T cells to produce cytokines such as IFN_Ɣ_ and IL-2 varied depending on individual pairings. Given the diversity of pathogen transmission routes, cellular trophism, and methods of infectious spread, incorporation of particular adjuvants capable of modulating specific features of the vaccine-induced immune response will likely result in the development of more effective vaccines. For instance, formulations containing adjuvants that skew the antibody response toward neutralisation, might be preferred for viruses which shed infectious virions, while adjuvants that induce ADCC- and neutralisation-capable antibodies such as LQ and LMQ might be preferred for viruses which spread *via* cell-to-cell contact. Depending on the cell type infected by individual pathogens (neurons, cardiomyocytes, etc.), the induction of cytotoxic T cells and ADCC-inducing antibodies by a vaccine candidate might result in undesired tissue damage. Comparison studies such as these provide an opportunity to improve the way that vaccines are developed.

The vaccine formulations used in our studies contained a pre-fusion molecular clamp-stabilized MERS-CoV S protein subunit (9). The molecular clamp is used to stabilize the spike glycoprotein in the authentic pre-fusion conformation that preserves neutralising epitopes present on the virion surface, which become hidden following viral fusion (11). Consequently, neutralising antibody responses can block the association of virions with host cells to prevent infection. This technology has been validated in several vaccination studies in humans and mice for SARS-CoV-2 (mice and humans) (10, 11, 25), Influenza A (mice) (26), and respiratory syncytial virus (RSV) (mice) (27). While the findings of this study are relevant for MERS-CoV SClamp, it would be anticipated that similar trends will extend to other antigens. Other studies have identified that different antigens when formulated with the same adjuvant can elicit immune responses of differing magnitudes (27). Biophysical properties of proteins can impact their interaction with the adjuvant, in turn affecting the stability and biological activity of the vaccine formulation (28, 29). It is also possible that particular adjuvants might affect protein structure and stability (28, 29). While it is a limitation of this study that we have only evaluated immune responses to a single antigen, in doing so, we have provided a useful workflow that could be applied to identify ideal antigen-adjuvant pairings.

Adjuvant technology has been revolutionized over the past two decades. In-depth understanding of the role of adjuvants in activating the innate immune system, combined with systems vaccinology approaches have led to the development of next-generation, novel adjuvants. While top-down approaches such as those used in this study are undoubtedly useful, a fundamental understanding of the mechanisms by which different adjuvants induce immune activation is lacking. For instance, in this study we demonstrated that the LMQ adjuvant significantly boosted the antibody response and biased it toward IgG2c production. LMQ are neutral liposome-based formulations containing cholesterol, DOPC, and QS21, a saponin adjuvant that activates antigen presenting cells such as dendritic cells and promotes their secretion of Th1 cytokines (16). LMQ also contains the synthetic TLR4 agonist 3D6AP (30). While these processes have been noted to occur, it is not clear how they combine to influence B cell maturation and isotype switching. Studies to investigate adjuvant mechanisms of action should also be a priority.

Mice are the preferred model for evaluating immunogenicity of novel vaccine formulations during the early stages of vaccine development. Aside from differences in B and T cell repertoires, many elements of mouse and human innate and adaptive immune systems are functionally similar. Adjuvants included in modern vaccines activate innate immunity through *via* pattern recognition receptors and contribute to the induction of adaptive responses. However, for pattern recognition receptors such as TLR, expression in different cell types or subsets (31, 32) as well as TLR4 or TLR8 ligand specificity (33, 34) can limit the predictive value of the mouse model. In this study, squalene in water emulsion and immunostimulatory compounds such as QS21 and 3D6AP have been selected for their ability to stimulate immune responses in mice and humans (13, 30, 35–39). This approach mitigates risks associated with down-selecting suitable antigen/adjuvant combinations before entering clinical studies.

This work uses a prefusion stabilized MERS SClamp protein subunit to compare the impact that various vaccine adjuvants have on features of the antigen-specific immune response. In doing so, we have highlighted that individual adjuvants can skew features of the immune response. Our workflow should serve as a template for rational adjuvant selection in vaccine development. Finally, the adjuvant selection process described in this study is made possible because open access adjuvant technologies available or soon to become available at clinical grade combined to formulation know how allows for rapid evaluation of novel vaccine antigens and progression towards clinical studies. The approach will be key for the development of novel vaccines during future pandemics.

## Materials and methods

### Mice

Wild type (WT) C57BL/6 mice were purchased from Charles River. All mice were housed at University of Geneva (Switzerland) animal facility. BALB/c WT mice were purchased from Australian Resource Centre and housed at the Australian Institute for Bioengineering and Nanotechnology at the University of Queensland, Australia. Mice greater than 8 weeks of age were used in all experiments. The number of mice in each treatment group has been indicated in the figure legends. No mice were excluded based on pre-established criteria and randomization was applied immediately prior to treatments in vaccination experiments. Experiments were conducted at either the Vaccine Formulation Institute, Geneva, Switzerland, or at The University of Queensland, Brisbane, Australia. Experiments were conducted in accordance with either the Swiss Federal Animal Protection Act, or procedures approved by the University of Queensland Animal Ethics Committee.

### Cell lines

Mouse LA-4 (Lung Adenoma) originally obtained from the ATCC (CCL-196) were cultured in Ham’s F-12K (Kaighn’s) Medium (ThemoFisher Scientific, Waltham, MA, USA), supplemented with 20 mM HEPES, 100 U/mL Penicillin Streptomycin (ThemoFisher Scientific, Waltham, MA, USA), 10% (v/v) Fetal Bovine Serum (FBS) (ThemoFisher Scientific, Waltham, MA, USA), 0.08% (w/v) Sodium Bicarbonate (ThemoFisher Scientific, Waltham, MA, USA) 1 x MEM Non-essential Amino Acids (ThemoFisher Scientific, Waltham, MA, USA), 1 x GlutaMAX (ThemoFisher Scientific, Waltham, MA, USA), and 600µg/mL G418 (ThemoFisher Scientific, Waltham, MA, USA). LA-4 cells (P4) were transfected with pNBF MERS S FL (2500 ng per well of 6 well plate) using Lipofectamin 2000 in accordance with the manufacturer’s protocol. FK12 media, prepared as described above, was replaced every 2 to 3 days. FACS sorting was performed 4 consecutive times to select for positive transfectants (>90% of cells following the 4^th^ sort). ExpiCHO cells, originally obtained from ThermoFisher, were cultured in ExpiCHO-S Expression Medium (ThemoFisher Scientific, Waltham, MA, USA).

### Antigen preparation

To express the prefusion MERS-S ectodomain, codon-optimized MERS-CoV KFU-HKU-13 strain S (amino acids 1-1297, GenBank ID: AHX00711.1) with variations generated using primers containing overlapping sequence by PCR mutagenesis using Phusion polymerase (New England Biolabs, Ipswich, MA, USA). These amplicons were introduced into a mammalian expression vector upstream of the clamp trimerization motif. Variable domains of heavy and light chains of G4 (anti-MERS S) (40), and anti-clamp HIV1281 (41), were clones into the mammalian expression vector pNBF-Hv or pNBF-lV in-frame with IgK signal peptide.

The ExpiCHO-S expression system (ThemoFisher Scientific, Waltham, MA, USA) was used for transient spike protein and antibody expression. ExpiCHO cells were transfected with DNA in accordance with the manufacturer’s instructions and cultured for 5 to 7 days. Culture supernatants were then isolated for protein purification. MERS-SClamp protein was purified using immunoaffinity chromatography on an ÄKTA pure protein purification system (Cytiva, Marlborough, MA, USA). This was done using an immunoaffinity chromatography column prepared in-house with HIV1281 anti-clamp antibody (coupled to 1 or 5 mL HiTrap-NHS-activated HP columns (Cytiva, Marlborough, MA, USA). ExpiCHO expression cultures were centrifuged at 4,000 g for 10 minutes at 4°C. Filtered supernatants were then added to an anti-clamp protein affinity column to purify clamp-stabilised proteins or protein A HP column (Cytiva, Marlborough, MA, USA) to purify antibodies. Following purification, eluates from culture supernatant were neutralized to neutral pH and buffer-exchanged to PBS using Amicon Untra-4 or Ultra-15 centrifuge filter units (Merck, NJ, USA). Protein concentration was determined using a NanoDrop One (ThemoFisher Scientific, Waltham, MA, USA) or using a BCA assay (ThemoFisher Scientific, Waltham, MA, USA) in accordance with the manufacturer’s instructions.

### Adjuvant preparation and formulation characterisation

Aluminium hydroxide gel (Alhydrogel 2%) was purchased from Croda. SWE adjuvant (Squalene-in-water emulsion) was manufactured at SEPPIC as previously described (42). SQ, SMQ, LQ and LMQ adjuvant formulations were manufactured at the Vaccine Formulation Institute as previously described (36). Briefly, SQ adjuvant was prepared by mixing a solution of QS21 (Desert King International, CA, USA) in PBS with squalene-in-water emulsion, containing cholesterol (Merck-Sigma C1231, USA). SMQ adjuvant was prepared in the same way but with incorporation of the synthetic TLR4 agonist 3D-(6-acyl) PHAD (3D6AP, Merck-Avanti 770050, USA). LQ adjuvant was prepared by adding a solution of QS21 (Desert King International, CA, USA) in PBS to neutral liposomes composed of 1,2-dioleoyl-sn-glycero-3-phosphocholine (DOPC) and cholesterol as lipids. LMQ adjuvant was prepared like LQ but with incorporation of 3D6AP. Full adjuvant physicochemical characterization was performed on all formulations containing adjuvants mixed 1:1 with MERS SClamp antigen before injection in mice. Each injectable dose of LQ, SQ, LMQ and SMQ contained 2 µg of the TRL4 agonist 3D6AP and/or 5 µg of QS21 saponin as described previously (36). For the SWE, SQ and SMQ oil in water emulsions each injectable dose of adjuvant contained 1mg squalene (36).

### Antigen integrity analysis

Antigen integrity Nunc-Immuno Maxisorb™ plates (ThermoFisher Scientific, Waltham, MA, USA) coated with capture monoclonal anti-clamp IgG2a at 4 µg/mL were incubated for 2 hours with PBS containing 2% bovine serum albumin (BSA) (w/v) (Sigma, MO, USA), washed (PBS containing 0.05% Tween20 [v/v]), and incubated with dilutions of adjuvanted formulations for 1 hour at 37°C. Plates were then washed twice and incubated for 1 hour with the human anti-MERS m336 monoclonal antibody (43). Plates were then washed and incubated with a polyclonal goat anti-human IgG coupled to peroxidase (Sigma A0170, MO, USA). Plates were then washed 4 times before addition of o-phenylenediamine dihydrochloride (OPD) peroxidase substrate. reaction was stopped with the addition of a 25% sulfuric acid (v/v) solution. Absorbance was measured at 492 nm using a microplate reader (Tecan, Switzerland).

### Mouse immunizations, sample collection and preparation

Injectable formulations were prepared at either a high (5 µg) or low antigen dose (1 µg) with the following adjuvants and buffers; AIOH in WFI and HEPES, SWE, SQ, and SMQ in PBS (pH 7.2) with no Ca^2+^, Mg^2+^ or NaCl, LQ and LMQ in PBS (pH 6.3) with no Ca^2+^ or Mg^2+^. Mice were immunized twice on days 0 and 21 with 50 µL of vaccine or controls (either excipient alone or antigen + excipient, 50 µL/mouse) intramuscularly with the two doses administered into alternating hind legs. For T cell analysis, spleens were collected from 2 mice for the excipient group and 4 mice for groups with antigen on day 35. For antibody isotype analysis, blood was collected from mice on day 35. Blood was centrifuged at 10,000 g for 10 minutes and the serum layer was collected before being stored at -80°C prior to analysis.

### Pseudovirus neutralization assay

MERS-CoV pseudoviruses were generated as previously described (44, 45). Briefly, 2 x 10^6^ HEK293T cells were plated in DMEM 10% FCS (D10) media in a 10 cm^2^ dish and incubated overnight at 37°C with 5% CO_2_. Cells were transfected with 1 µg p8.91 (encoding HIV-1 *gag-pol* genes), 1.5 µg pCSFLW (encoding firefly luciferase) and 1 µg of MERS S glycoprotein (residues 1-1353, GenBank: AHX00711.1) expressed under CMV promoter in pNBF. Transfection was done using lipofectamine transfection reagent (Invitrogen) as per manufacturer’s instructions. Cells were incubated overnight at 37°C with 5% CO_2_. The next day, the media was replaced with 7 mL D10 and incubated for a further 24 hours. Virus was harvested three times 12, 24 and 36 hours later. Pooled harvests were centrifuged at 1,300 x g for 10 minutes at 4°C to remove cellular debris before aliquoting and storage at -80°C.

To measure MERS-CoV pseudovirus titer, Huh-7.5 cells were plated at 2 x 10^4^ cells per well of a white Nunc MicroWell™ 96-well plate in D10 media and incubated at 37°C with 5% CO_2_ overnight. The following day, MERS-CoV pseudovirus was tittered on target cells 5-fold in D10 media and incubated at 37°C with 5% CO_2_ for 3 days. Firefly luciferase signal was measured by discarding supernatant from cells and adding 50 µl/well of a 1:1 mix of serum-free DMEM and Bio-Glo Luciferase Assay System reagent (Promega) for 10 minutes. Relative light unit (RLU) readings were quantified using a VarioSkan LUX luminescence plate reader (ThermoFisher).

To measure virus neutralization by serum samples, target Huh-7.5 cells were plated and incubated as described for the pseudovirus titer assay. The next day, heat-inactivated serum samples were serially diluted in D10 media in triplicate before adding an equal volume of MERS-CoV pseudovirus at a dilution that would yield a signal of ~2 x 10^6^ RLU. Serum and virus samples were then incubated for 1 hour at 37°C before adding onto Huh-7.5 cells and incubating for a further 72 hours at 37°C with 5% CO_2_. Firefly luciferase reporter activity was quantified as previously described for pseudovirus titer assay.

### Antibody isotype analysis

Anti-MERS Ig responses were measured in mouse sera using ELISA. Nunc-Immuno Maxisorb™ plates (ThermoFisher Scientific, MA, USA) were coated directly with MERS SClamp antigen at 0.25 µg/mL in PBS overnight at 4°C, blocked for 2 hours with PBS containing 2% BSA (w/v) (Sigma, MO, USA), washed with PBS containing 0.05% Tween20 (v/v), and incubated with 4x serial dilutions of mouse serum samples diluted 1/400 in PBS containing 0.05% BSA. Plates were incubated for 90 minutes, washed, and further incubated for 1 hour with anti-mouse antibodies coupled to HRP (SouthernBiotech, AL, USA) total Ig, IgG1, IgG2b or IgG2c. Plates were then washed before addition of 3, 3’, 5, 5’-Tetramethylbenzidine (Sigma, MO, USA) for 4 minutes and the reaction was stopped by adding 1 M sulfuric acid. Absorbance was then measured at 450 nm using a microplate reader (Tecan, Switzerland). The calculation of percentages shown in **Figure 2D** was based on the values displayed in **Figures 2A–C**. Percentages were calculated using the following % = 100 x (m/b) where m = the group average for either IgG1, IgG2b, or IgG2c, and b = the sum of each group’s average (IgG1 + IgG2b + IgG2c).

### ADCC assay

LA4 cells transfected to stably express MERS S protein antigen, were washed with PBS, and treated with 0.05% Trypsin EDTA (ThemoFisher Scientific, Waltham, MA, USA). Once cells detached they were resuspended with PBS and centrifuged at 4,000 g for 4 minutes. The supernatant was discarded, the cell pellet was resuspended in 1 mL of PBS containing 0.5 µM Carboxyfluorescein succinimidyl ester (CFSE) (ThemoFisher Scientific, Waltham, MA, USA), and incubated at 37°C for 15 minutes. Cells were then washed by resuspension in PBS containing 5% FBS (FACS Buffer) and centrifuged at 4,000 g for 4 minutes. Cell pellets were resuspended in 1 mL of FACS buffer and counted using a Coutess II (ThemoFisher Scientific, Waltham, MA, USA). Cells were then added to wells of a 96 well plate (2.0 x 10^4^ cells/well).

A spleen was isolated from a treatment-naïve female BALB/c mouse, mechanically dissociated through a 0.4 µm cell filter, resuspended in PBS, and centrifuged at 4,000 g for 4 minutes. The supernatant was discarded, and the cell pellet was resuspended in 1 mL of red blood cell (RBC) lysis buffer (ThemoFisher Scientific, Waltham, MA, USA) and incubated for 2 minutes at room temperature. RBC lysis was stopped by the addition of FACS buffer, cells were then centrifuged at 4,000 g for 4 minutes, and the cell pellet resuspended in 1 mL of FACS buffer. Splenocytes were then counted using a Coutess II (ThemoFisher Scientific, Waltham, MA, USA). Splenocytes were then diluted and added (1.0 x 10^6^ cells/well) in 5 µL of FACS buffer to LA4 cells. Serum samples were diluted to 1/10^3^ in and the total volume increased to 250 μL with FACS Buffer. Plates were then incubated at 37°C for 4 hours. Following incubation, plates were transferred onto ice, and 7AAD (2 M) was added to each well with a final volume of 100 µL (24).

### Flow cytometry and cell sorting

For analysis of ADCC, unstained LA4 cells, or samples stained with CFSE-alone, or 7AAD-alone were used to calibrate compensation. Samples were analyzed by first gating on cells of LA4 cell morphology and then by evaluating the proportion of CFSE+ (LA4 cells) 7AAD+ (dead cells) cells. %Specific LA4 cell death was calculated using the formula (x – y)/(100-y) x 100, where x represents the % of CFSE+7AAD+ cells within experimental samples and y represents the % of CFSE+7AAD+ cells within the excipient control condition. Data acquired on either a Fortessa or FACSMelody (Becton Dickinson, NJ, USA), and analyzed using FlowJo v10 (Becton Dickinson, NJ, USA).

For serum antibody labelling of LA4 cells expressing MERS antigen, cells were washed with PBS, and treated with 0.05% Trypsin EDTA (ThemoFisher Scientific, Waltham, MA, USA). Once cells detached they were resuspended with PBS and centrifuged at 400 g for 4 minutes. The supernatant was discarded and the cell pellet was resuspended in 1 mL of FACS Buffer. The cells were counted using a Countess II (ThemoFisher Scientific, Waltham, MA, USA) and cells were added to wells of a 96 well plate (2.0 x 10^4^ cells/well) and combined with serum at the specified dilutions in a final volume of 200 mL. Cells were incubated for 30 minutes at 4°C then, centrifuged at 400 g for 4 minutes. The supernatant was discarded and the cells were washed with 200 mL of FACS buffer followed by centrifugation at 400 g for 4 minutes. The supernatant was discarded, and cells were then resuspended with FACS buffer (30 uL) containing anti-mouse IgG (RRID: AB_933619) PE (eBioscience™) diluted to 1/500 and Zombie Aqua diluted to 1/500 and incubated on ice for 30 minutes. Cells were then washed with FACS buffer (100 uL) and centrifuged at 400 g for 4 minutes. Cell pellets were resuspended with FACS buffer (100 uL). Unstained and single colour controls (Zombie Aqua and anti-mouse IgG PE) were included for compensation calculation.

For analysis of T cells response, splenocytes were isolated by crushing spleens through a 70 µm cell strainer, then washed with RPMI medium containing 1% penicillin/streptomycin, 20 mM HEPES + 1X MEM NEAA) followed by red blood cells separation using Lympholyte^®^-M cell separation medium (Cedarlane, Canada). Splenocytes were then washed with complete RPMI medium (RPMI with 1% penicillin/streptomycin, 20 mM HEPES + 1X MEM NEAA, 6% foetal calf serum and 50 µM 2–Mercapto-ethanol). Cells were seeded in a 96-well plate and stimulated with a pool of 20mer peptides with 10aa overlap at 1.5 µg/mL/peptide combined with 1 µg/mL anti-mouse CD28 (BD Biosciences) or medium containing only 1 µg/mL anti-mouse CD28 and incubated for 2 hours at 37°C. Cytokine secretion was blocked using BD GolgiPlug™ protein transport inhibitor and cells were further incubated for 16 hours at 37°C, 5% CO_2_. Cells were then placed on ice, transferred to V-bottom 96-well plates and stained for 15 minutes at 4°C using the LIVE/DEAD™ reagent (Life Technologies, CA, USA). Cell surface staining was performed with anti-CD3e-(clone 17A2)-FITC (eBioscience™) and anti-CD8a-(clone 53-6.7)-PerCP-Cy5.5 (BioLegend^®^) at 1:100 dilution and anti-CD4-(clone GK 1.5)-Pacific Blue at 1:1000 dilution for 15 minutes on ice. After washing, fixation and permeabilization using Cytofix/Cytoperm™ (Becton Dickinson), cells were incubated for 30 minutes at 4°C with anti-IL-2-(clone JES6-5H4)-APC at 1:100 dilution and anti-IFNγ-(XMG1.2)-PE (BD Pharmingen™) at 1:200 dilution for intracellular staining. Cells were washed 3 times with Perm/Wash buffer (Becton Dickinson), resuspended in 300 μL PBS and filtered prior acquisition on a LSR Fortessa™ flow cytometer (BD Biosciences). Compensation was performed using the Arc™ Amine Reactive Compensation Bead Kit (Invitrogen, CA, USA) and compensation matrices were calculated with the BD FACSdiva™ software (BD Biosciences). FACS data were analyzed using FlowJo software (FlowJo LLC, USA).

## Statistical analysis

Statistical analyses were conducted as follows: For analyses in **Figures 1A, B, A–C**, and **S2A** EC50 values were generated using Graphpad Prism version 9.3.1. Values were then log10 transformed and graphed for each group. In **Figures 1A, B**, **2A–C**, **3D**, and **S2A**, statistical significance was tested between groups using Kruskal-Wallis tests with Dunn’s post-test. Regression analyses performed in **Figures S2B** and **S3C** were also performed using Graphpad Prism version 9.3.1 using values treated as described above. P>0.05 = ns, *P<0.05, **P<0.01, ***P<0.001, and ****P<0.0001.

## Data availability statement

The raw data supporting the conclusions of this article will be made available by the authors, without undue reservation.

## Ethics statement

The animal study was reviewed and approved by The University of Queensland Animal Ethics Committee.

## Author contributions

Conceptualisation: KJC, NC, PMD. Formal analysis: JO’D, VJ, CL, AI, KJC. Funding acquisition: KJC, NC, PMD, PRY, DW. Investigation: VJ, CL, AI, JO’D. Methodology: VJ, CL, AI, J O’D, JB, PRR. Supervision: KJC, NC, PRY, DW. Writing – original draft: JO’D, AI. Writing – reviewing & editing: JO’D, KJC, PMD, NC, VJ, CL. All authors contributed to the article and approved the submitted version.

## Funding

This work was supported by the National Health and Medical Research Development grant from the Australian Government (AP1156063) and the Coalition for Epidemic Preparedness Innovations (CEPI).

## Acknowledgments

This work was supported by the National Health and Medical Research Development grant from the Australian Government (AP1156063) and the Coalition for Epidemic Preparedness Innovations (CEPI). We thank Julia Lackenby for laboratory management at AIBN and the staff at the AIBN animal facility for providing technical guidance during the mouse experiments. KJC, DW and PRY report holdings in ViceBio Limited and have patents pending related to molecular clamp platform (AU 2018241252; BR112019019813.9; CA 3057171; CH 201880022016.9; EP 18775234.0; IN 201917038666; ID P00201909145; IL 269534; JP 2019-553883; MX/a/2019/011599; NZ 757178; KR 0-2019-7031415; SG 11201908280 S; US 16/498865). The development of the saponin-containing adjuvants is supported by a grant from the Bill and Melinda Gates Foundation (INV001759) to VFI. The authors also wish to thank Seppic for kindly providing the SWE adjuvant. CL, VJ, PMD, and NC are employees of VFI which has a licensing agreement with Seppic for the commercialisation of SWE under the trade name Sepivac SWE™. All remaining authors have nothing to disclose.

## Conflict of interest

KJC, DW and PRY report holdings in ViceBio Limited and have patents pending related to molecular clamp platform (AU 2018241252; BR112019019813.9; CA 3057171; CH 201880022016.9; EP 18775234.0; IN 201917038666; ID P00201909145; IL 269534; JP 2019-553883; MX/a/2019/011599; NZ 757178; KR 0-2019-7031415; SG 11201908280 S; US 16/498865). CL, VJ, PMD and NC are employees of VFI which has a licensing agreement with Seppic for the commercialisation of SWE under the trade name Sepivac SWETM.

The remaining authors declare that the research was conducted in the absence of any commercial or financial relationships that could be construed as a potential conflict of interest.

## Publisher’s note

All claims expressed in this article are solely those of the authors and do not necessarily represent those of their affiliated organizations, or those of the publisher, the editors and the reviewers. Any product that may be evaluated in this article, or claim that may be made by its manufacturer, is not guaranteed or endorsed by the publisher.
