# Supplementary material for: Characterization and comparison of novel adjuvants for a prefusion clamped MERS vaccine
Source: Front Immunol. 2022 Sep 2;13:976968. doi: 10.3389/fimmu.2022.976968 (PMC9478912; doi:10.3389/fimmu.2022.976968)
Supplement: Supplementary file 1 [file DataSheet_1.docx]

**Supplemental Material: Characterization and comparison of novel adjuvants for a prefusion clamped MERS vaccine**

Jake S. O’Donnell, Ariel Isaacs, Virginie Jakob, and Celia Lebas et al.

**Figure S1. (A)** Using the same experiment protocol as Figure 1A, ELISAs showing anti-MERS IgG titer of serum from mice treated with high dose (5µg/mouse) MERS SClamp antigen +/- adjuvants. Kruskal-Wallis with Dunn’s test, ns = P>0.05, and **P<0.01. Experiment completed once, n = 3 – 6 mice/group. **(B)** From the same experiment as Figure 1A and Figure 1B, linear regression of antibody titers and pseudovirus neutralization IC_50_, with line of best fit, r^2^, and exact *P* value also displayed.

**Figure S2. (A)** Titration of serum from MERS SClamp-immunized mice or those with +SWE or +SQ adjuvants with LA4 MERS antigen-expressing cells (LA4 MERS) with antibody binding labeled with anti-mouse PE. Representative flow cytometry histograms of PE mean florescence intensity (MFI). The median No Serum control histogram has been included in all graphs (grey). **(B)** Data summary presented as mean of 3 technical replicates +/- SEM. **(C)** From Figure 2C and Figure 3D, linear regression analysis of the IgG2c titers for each experimental group plotted against normalized % specific LA4 MERS cell death, n = 3 – 6 mice/group. Line of best fit, exact r^2^, and *P* values have also been displayed.

**Figure S3.** Splenocytes were isolated from C57/BL6 mice immunized with the same experimental groups as in Figure 4, however, with immunizations containing a higher antigen dose (5 ug/mouse). Splenocytes were isolated and incubated with or without MERS SClamp antigen peptides in the presence of protein transport inhibitors and analysed by flow cytometry. Using the same gating strategy defined in Figure 4B, **(A)** Data summary for IFN_Ɣ_ and IL-2 staining among CD4^+^ T cells treated as described. **(B)** Data summary for IFN_Ɣ_ and IL-2 staining among CD8^+^ T cells treated as described. Individual data points for each mouse have been presented, n = 2 – 4/group.

**Table S1**. Biophysical properties of antigen/adjuvant formulations were measured at day 1, day 7 and day 28. Findings were recorded as passed (P) if the following conditions were met: Particle size values within the expected range of 130-150; Polydispersity Index values below 0.15 indicating a monodisperse size distribution; Zeta potential between -10 and -40mV as typical for these adjuvants; pH values 6.5-7.0 for SWE, SQ, SMQ or 6.0-6.5 for LQ and LMQ; Squalene content 20 (+/- 0.2) mg/ml ; Cholesterol content: 0.4 (+/- 0.1) mg/ml for SQ/SMQ and 0.2 (+/- 0.05) mg/ml for LQ/LMQ; DOPC content: 1.0 (+/- 0.15) mg/ml for LQ/LMQ; 3D6AP content: 40 (+/- 15) μg/ml; QS21 content: 100 (+/- 20) μg/ml; Antigen integrity ELISA with JC57 mAb no more than 50 decrease in signal.

Figure S1

Figure S2

Figure S3

Table S1.

|  | SWE | SQ | SMQ | LQ | LMQ |
| --- | --- | --- | --- | --- | --- |
| Particle size | P | P | P | P | P |
| Polydispersity | P | P | P | P | P |
| Zeta Potential | P | P | P | P | P |
| pH | P | P | P | P | P |
| Squalene content | P | P | P | NA | NA |
| Cholesterol content | NA | P | P | P | P |
| DOPC content | NA | NA | NA | P | P |
| 3D6AP content | NA | NA | P | NA | P |
| QS21 Content | NA | P | P | P | P |
| Antigen integrity | P | P | P | P | P |
